# Proteomics analysis reveals that the proto-oncogene eIF-5A indirectly influences the growth, invasion and replication of *Toxoplasma gondii* tachyzoite

**DOI:** 10.1186/s13071-021-04791-6

**Published:** 2021-05-26

**Authors:** Xinchao Liu, Chunjing Li, Xiaoyu Li, Muhammad Ehsan, Mingmin Lu, Ke Li, Lixin Xu, Ruofeng Yan, Xiaokai Song, XiangRui Li

**Affiliations:** 1 Anhui Province Key Laboratory of Animal Nutritional Regulation and Health, College of Animal Science, Anhui Science and Technology University, Fengyang, 233100 People’s Republic of China; 2 MOE Joint International Research Laboratory of Animal Health and Food Safety, College of Veterinary Medicine, Nanjing Agricultural University, Nanjing, 210095 People’s Republic of China; 3 State Key Laboratory of Veterinary Etiological Biology, Key Laboratory of Veterinary Parasitology of Gansu Province, Lanzhou Veterinary Research Institute, Chinese Academy of Agricultural Sciences, Lanzhou, 730046 Gansu People’s Republic of China; 4 Poultry and Poultry Diseases Institute, Yunnan Animal Science and Veterinary Institute, Kunming, 650224 People’s Republic of China

**Keywords:** *Toxoplasma gondii*, EIF-5A, Invasion, Replication

## Abstract

**Background:**

The proliferative stage (tachyzoite) of *Toxoplasma gondii* (*T. gondii*) is critical for its transmission and pathogenesis, and a proto-oncogene eukaryotic translation initiation factor (eIF-5A) plays an important role in various cellular processes such as cell multiplication.

**Methods:**

We performed a proteomic study to evaluate the specific roles of eIF-5A involved in invasion and replication of *T. gondii*, and both in vivo and in vitro trials using eIF-5A-interfered and wild tachyzoites were performed to verify the proteomic results.

**Results:**

The results of our study showed that *T. gondii* eIF-5A affected tachyzoite growth and also participated in the synthesis of proteins through regulation of both ribosomal and splicing pathways. Inhibition of eIF-5A in *T. gondii* resulted in the downregulated expression of soluble adhesions, such as microneme protein 1 (MIC1) and MIC4, which in turn decreased the parasite population that adhered to the surface of host cells. The reduced attachment, combined with lower expression of some rhoptry proteins (ROPs) and dense granule antigens (GRAs) involved in different stages of *T. gondii* invasion such as ROP4 and GRA3, ultimately reduce the invasion efficiency. These processes regulated by eIF-5A eventually affect the replication of tachyzoites.

**Conclusions:**

Our findings showed that eIF-5A influenced tachyzoite survival and was also involved in the process of parasite invasion and replication. These results will provide new clues for further development of targeted drugs to control *T. gondii* infection.

**Graphic abstract:**

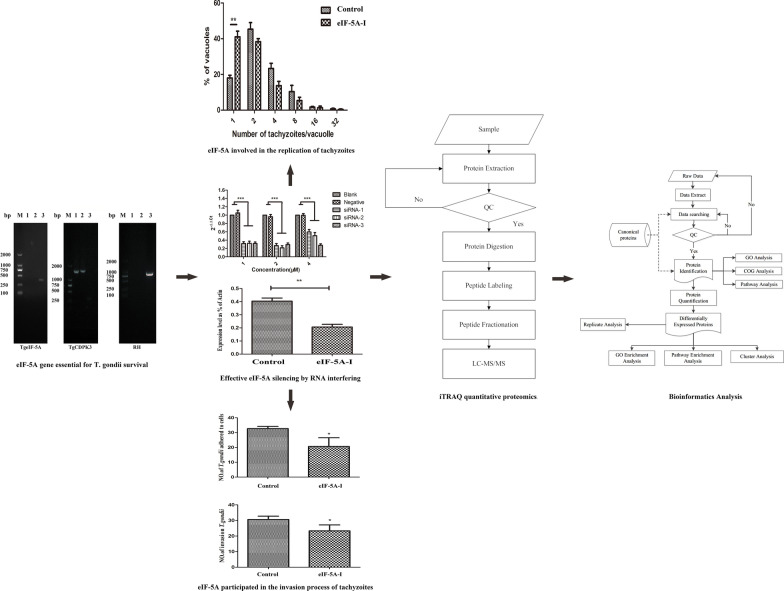

**Supplementary Information:**

The online version contains supplementary material available at 10.1186/s13071-021-04791-6.

## Background

Proto-oncogenes are normal cellular genes involved in the regulation of proliferation and differentiation. On activation, proto-oncogenes convert into oncogenes, free from regulatory constraints on cell growth and division. Previous studies have suggested that eukaryotic elongation factor-2 (eEF-2), an oncogene, is over-expressed in cancer cells and participates in proliferation, apoptosis and invasion signaling pathways [1–3]. Deregulated oncogenic *Myc* contributes to cancer cell proliferation and drives tumor genesis [4]. Sequence homology analysis of the *T. gondii* genome has shown the presence of proto-oncogenes; however, little is known about the relationship between these genes and the replication of *T. gondii*.

*T. gondii* is an obligate intracellular parasite with three infectious stages in its life cycle: tachyzoite, bradyzoite and oocyst [5]. Tachyzoites are characterized by a rapidly growing stage during the acute phase of infection in host cells, perpetuating the lytic cycle via host cell invasion, intracellular replication and parasite egress. Replication is the most critical process in parasite physiology and pathogenesis of *T. gondii* [6, 7], and host cell invasion is the prerequisite for tachyzoite replication. Invasion of host cells by *T. gondii* is a multistep process accompanied by secretion of numerous regulated proteins from three distinct parasite organelles called micronemes, rhoptries and dense granules [8]. The first specialized secretory organelle is the microneme, discharging a variety of proteins that allow the parasite to reach putative host cells and form an intimate binding interface junction connecting host cell receptors and parasite adhesion proteins [9, 10]. Moreover, the concentration of rhoptries and dense granules has been associated with the formation of a specialized parasitophorous vacuole (PV) [11, 12], which results in parasite internalization through the endocytic pathway [6].

Host cell invasion by *T. gondii* is an active process accomplished within a few seconds after parasite–host cell interaction [13]. Once a tachyzoite enters a host cell, the replication linked to acute virulence starts, and the parasite multiplies asexually through an internal budding mechanism called endodyogeny, in which two daughter parasites are developed. The daughter budding is a highly coordinated phenomenon in all four stages: DNA replication, chromosome segregation, nuclear division, and finally cytokinesis or budding [14]. Furthermore, a tachyzoite accumulates 64–128 daughter parasites over 2 to 3 days with a generation time of 6 to 8 h before lysing the host cell to infect neighboring cells [6]. This rapid tachyzoite division is the underlying mechanism of pathogenesis, and despite this importance, relatively few molecules involved in replication have been characterized to date.

Initiation, elongation, termination and recycling are the four stages of protein synthesis. During the initiation phase, the small ribosomal subunits associated with mRNA locate the start codon for the open reading frame, and eukaryotic translation initiation factor 5A (eIF-5A) is one of the eukaryotic translation initiation factors required for the selection of the start codon, playing an essential role in cell proliferation [15]. Two forms of eIF-5A (eIF-5A1 and eIF-5A2) are expressed in both yeast and humans, with 80–90% shared amino acid sequence identity within each organism [16]. Leading researchers have reported that eIF-5A1 is expressed in all mammalian cells, and the mRNA of eIF-5A1 was found to be under the control of the *Myc* oncogene [17, 18]. In contrast, the expression of eIF-5A2 in mammalian cells was too low to detect, and the mRNA was detected only in specific tissues [18]. Consequently, the overexpression of eIF-5A2 has been associated with many cancer types, to the extent that it was proposed as a candidate oncogene [19, 20]. In *Saccharomyces cerevisiae*, the growth was arrested when eIF-5A was disrupted or substituted [21]. In various human cancer cell lines, the inhibitors of deoxyhypusine synthase (DHS) and deoxyhypusine hydroxylase were found to be involved in the formation of hypusine, and played a vital role in the antiproliferative effects [22, 23]. These studies collectively demonstrate that the eIF-5A gene contributes to cell proliferation, and appears to be an essential factor for yeast growth. However, the biological function of eIF-5A (TGME49_051810) in *T. gondii* tachyzoite cell invasion and replication has yet to be discovered. In the present study, we focus on the regulatory roles of eIF-5A in *T. gondii* invasion and replication. Our results provide a potential source for understanding the role of eIF-5A during *T. gondii* pathogenesis, which will aid in controlling toxoplasmosis in the near future.

## Methods

### Ethics statement

The research was performed following the guidelines of the Animal Ethics Committee, Nanjing Agricultural University, China. All of the experimental protocols were authorized by the Science and Technology Agency of Jiangsu Province. The approval ID is SYXK (SU) 2010–0005.

### Parasites and animals

RH strain tachyzoites were maintained in monolayers of a human foreskin fibroblast (HFF) cell line maintained with Dulbecco’s modified Eagle’s medium (DMEM; Invitrogen) containing 10% fetal bovine serum (FBS, Gibco, China) and 1% penicillin–streptomycin (Gibco, China).

Eight-week-old female Sprague Dawley (SD) rats, used for obtaining antibodies, and 5-week-old female BALB/c (18-22 g) mice, used in the virulence assay, were purchased from the Institute of Comparative Medicine, Yangzhou University (Yangzhou, China), and kept in a specific-pathogen-free (SPF) environment.

### Production of antibodies

The prokaryotic expression vector pET-32a (+) was utilized to produce the recombinant protein of *T. gondii* eIF-5A (rTgeIF-5A). The TgeIF-5A open reading frame (ORF) was amplified by polymerase chain reaction (PCR) from *T. gondii* cDNA with the oligonucleotides summarized in Additional file 1: Table S1 in the supplemental material. The plasmid was built using double-enzyme digestion. After expression and purification, the rTgeIF-5A protein emulsified with Freund’s adjuvant (Sigma, USA) was injected into two SD rats, followed by four boosters at 1-week intervals. Subsequently, the sera of the immunized rats were collected and validated by Western blot as described in a previous study, and the antibody against *T. gondii* was obtained in the previous study and stored in our laboratory [24].

### Plasmid and mutant strain construction

The plasmid pSAG1-CAS9-TgU6-sgRNA (UPRT) and pUPRT-*DHFR*-D were provided by Professor Bang Shen, Huazhong Agricultural University, PR China. The primers used in this study are listed in Additional file 2: Table S2. The plasmids were constructed as described by Shen et al. [25]. In addition, to ensure the accuracy of experimental operation, a nonessential gene CDPK3 knockout strain was generated together [26]. Briefly, pCRISPR-eIF-5A was constructed by changing the sgRNA of plasmid pSAG1-CAS9-TgU6-sgRNA (UPRT) to the specific sgRNAs described in Table S2 through a Q5 Site-Directed Mutagenesis Kit (New England Biolabs, USA). The homology template plasmids for deleting the target sequences peIF-5A-DHFR were generated by flanking the 5′ and 3′ ends of the eIF-5A coding regions surrounding the pyrimethamine-resistant DHFR* cassette using a ClonExpress^®^ MultiS one-step cloning kit (Vazyme Biotech, China). Mutations of the plasmids were identified by restriction enzyme digestion and confirmed by nucleotide sequencing together with the homology template plasmids. After identification, the plasmids were purified using the Endo-Free Plasmid Maxiprep kit (OMEGA, USA).

To introduce the pCRISPR-eIF-5A along with peIF-5A-DHFR into the parasites, electroporation was used. After 3 days of introduction, 1 μM pyrimethamine (APExBio, USA) was used to select the parasites with the correct replacement of DHFR* for 7 days, and the genomic DNA was then extracted. The knockout clones were identified by PCR 1, 2 and 3 to confirm the presence of positive mutant (Primers listed in Additional file 2: Table S2). Limiting dilution was then performed in 96-well plates to obtain single-cell clones, which was further confirmed by PCR and nucleotide sequencing. The eIF-5A knockout strains were collected and cultured for subsequent experiments. In addition, the knockout strains of CDPK3, a known nonessential gene, were constructed together with eIF-5A as a control in this study.

### Obtaining the eIF-5A gene knockdown parasites

As eIF-5A is an essential constituent of cells and yeast, knockdown of eIF-5A provides an alternative method to continue the study. Three small interfering RNA (siRNA) targeting TgeIF-5A were designed and synthesized by Invitrogen (Shanghai, China) (Additional file 3: Table S3). Aliquots of 1 μM, 2 μM and 4 μM of each TgeIF-5A siRNA and negative control (stealth siRNA Negative Control Lo GC, Invitrogen) were transfected into 1 × 10^7^ tachyzoites using the Gene Pulser Xcell™ Electroporation System (Bio-Rad, USA) at settings of 50Ω, 1500 V, and 25 µF. Parasites were added to the monolayer HFF cells after transfection and monitored to determine the transfection efficiency at 24 h after electroporation by real-time PCR and Western blotting (Additional file 6: Method S1 and Additional file 7: Method S2).

### Quantitative proteomics

The siRNA with the best transfection efficiency in optimal concentration was used for transfection of tachyzoites. Twenty-four hours after transfection, the parasites were harvested for quantitative proteomics, while RH strain parasites were set as the control group. Isobaric labeling for relative and absolute quantitation (iTRAQ) analysis and bioinformatics analysis of proteins was carried out at Beijing Genomics Institute (BGI, Shenzhen, China). Briefly, proteins were extracted and quantified from three biological repeats of eIF-5A knockdown parasites and RH strain parasites, respectively. Trypsin Gold (Promega, Madison, WI, USA) was used to digest 100 μg protein of each sample. After digestion, peptides were dried and dissolved in 0.5 M triethylammonium bicarbonate (TEAB). The peptide labeling was performed using the iTRAQ Reagent 8-plex Kit, according to the manufacturer's protocol (AB Sciex, USA), and the labeled peptides were mixed and dried by vacuum centrifugation. The peptides separated by nano-high-performance liquid chromatography (Shimadzu, Japan) were subjected to tandem mass spectrometry (Q Exactive; Thermo Fisher Scientific, USA) for data-dependent acquisition (DDA) detection by nano-electrospray ionization.

### Database search and bioinformatics analysis

The raw data were converted into MGF format, and the exported MGF files were searched by a local Mascot server against the database. In addition, quality control (QC) was performed to determine whether a re-analysis step was needed. An automated software program, IQuant, was applied for the quantification of proteins. To assess the confidence of peptides, the peptide-spectrum matches (PSMs) were pre-filtered at a PSM-level false discovery rate (FDR) of 1%, after which the identified peptide sequences were assembled into a set of confident proteins. In order to control the rate of false positives at the protein level, a protein FDR at 1% was also estimated after protein inference. The proteins with fold change > 1.2 and Q-value < 0.05 were deemed differentially expressed proteins (DEPs).

The DEPs were functionally classified according to Gene Ontology (GO) annotation and enrichment analysis, involving molecular function (MF), cellular component (CC), and biological process (BP) categories, using Blast2GO. The pathways of the DEPs were predicted by the Kyoto Encyclopedia of Genes and Genomes (KEGG; http://www.kegg.jp/kegg/). The protein–protein interaction (PPI) network of the DEPs was constructed using the STRING database (version 11.0) and visualized by Cytoscape software. The expression pattern of ribosomal proteins was also performed for DEPs.

### Invasion assay

The efficiency of host cell invasion was assessed as described in a previous study [27], with the following modifications: The siRNA with the best transfection efficiency in optimal concentration was used for transfection of tachyzoites, while RH strain parasites were set as the control group. The eIF-5A knockdown parasites and the control parasites were added to HFF monolayers or fixed HFF monolayers after transfection, respectively. Then parasites were allowed to invade or adhere for 1 h at 37 °C and 5% CO_2_. After incubation at 37 °C, the parasites were fixed and saturated. The rat sera against *T. gondii* were used to label the parasite, and a Cy3-conjugated anti-rat antibody was used as the secondary antibody. The number of the invaded or adhered parasites in 10 arbitrarily selected fields was calculated and analyzed using GraphPad Prism 5.0 software (GraphPad Software, USA). Experiments were carried out three times independently.

### Plaque assay

The siRNA with the best transfection efficiency in optimal concentration was used for transfection of tachyzoites, while RH strain parasites were set as the control group. The eIF-5A knockdown parasites and the control parasites were plated onto HFF monolayers in 12-well plates and incubated undisturbed at 37 °C and 5% CO_2_ for 7 days. Then the cells were fixed with 1% paraformaldehyde and stained with crystal violet solution (Beyotime, China). A microscope (Olympus, Japan) was used to acquire the plaques images, and the change in plaque-forming efficiency was calculated by Image Pro Plus 6.0 software (Media Cybernetics, USA). Experiments were carried out three times independently.

### Replication assay in vitro

The siRNA with the best transfection efficiency in optimal concentration was used for the transfection of tachyzoites, while RH strain parasites were set as the control group. The eIF-5A knockdown parasite and the control parasites were added into 12-well plates seeded with HFF monolayers, and incubated at 37 °C and 5% CO_2_ for 1 day. One hundred PVs were then randomly selected, and the number of parasites contained in each vacuole was counted. Experiments were carried out three times independently. The replication was analyzed using GraphPad Prism 5.0 (GraphPad Software, USA).

### Virulence assay

The siRNA with the best transfection efficiency in optimal concentration was used to for transfection of tachyzoites, while RH strain parasites were set as the control group. The eIF-5A knockdown parasites and the control parasites were intraperitoneally injected into 10 BALB/c female mice (200 or 2000 parasites/mouse), each dose for 5 mice, respectively. The survival time of mice was monitored daily, and the survival curve was analyzed using GraphPad Prism 5.0 (GraphPad Software, USA). Experiments were carried out three times independently.

### Statistics

The data for each experiment were indicative of three individual experiments. Statistical comparisons between eIF-5A knockdown and control parasites were conducted using Student’s *t* test. To compare the transfection efficiency of each siRNA, two-way ANOVA with Bonferroni post hoc tests was used. Statistical analysis of the survival time of parasite burden in mice was conducted by log-rank test and Gehan-Breslow-Wilcoxon test using GraphPad Prism 5.0 (GraphPad Software, USA). Differences were regarded as statistically significant at *P* values < 0.05.

## Results

### eIF-5A gene essential for *T. gondii* survival

**Fig. 1:**
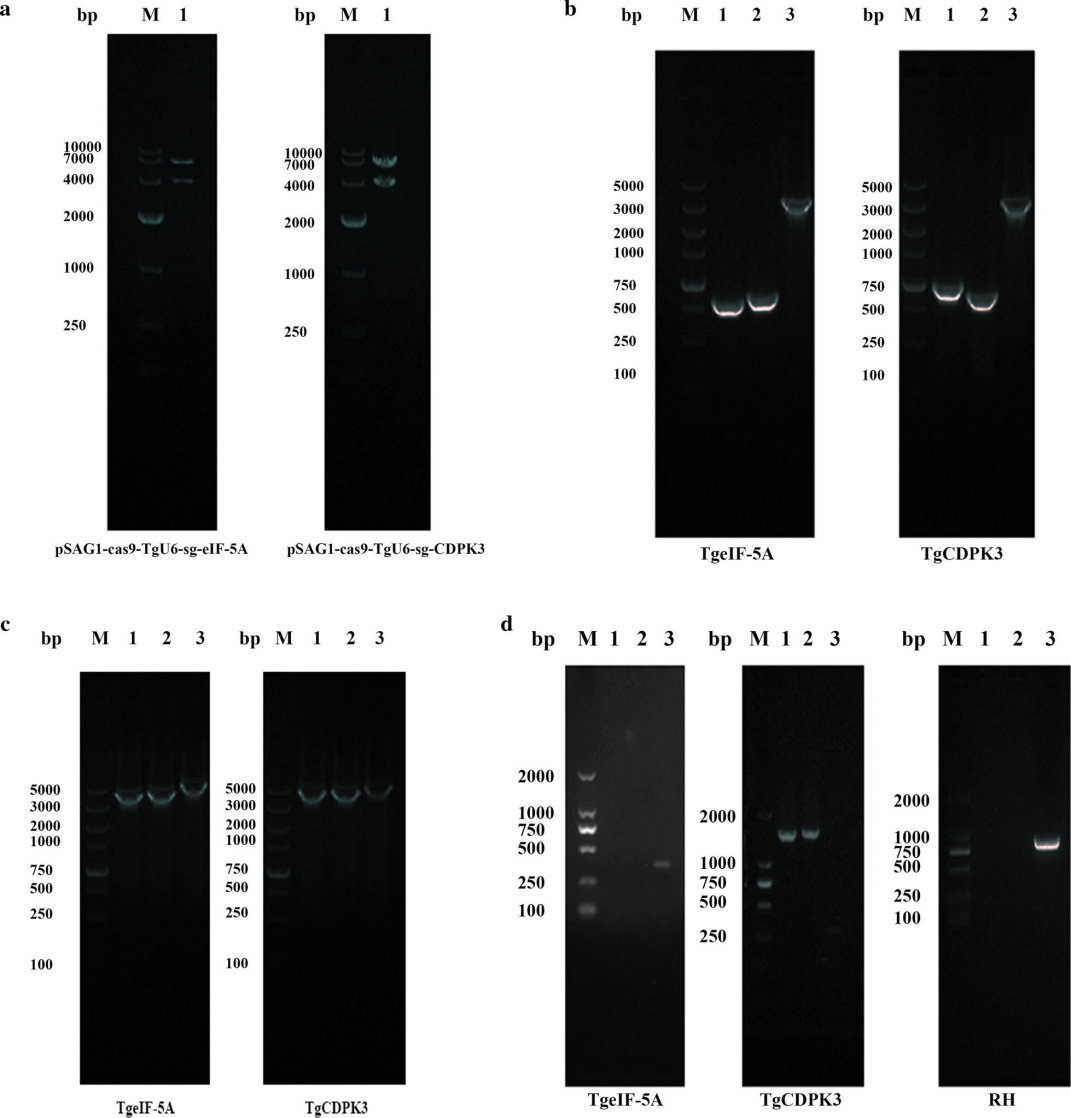
Construction of TgeIF-5A knockout mutant using CRISPR/Cas9. **a** Identification of the construction of pCRISPR-eIF-5A. (Lane M) DNA marker DL10000; (Lane 1) pSAG1-Cas9-U6-sg-eIF-5A digested by *Sal*I enzymes; (Lane 2) pSAG1-Cas9-U6-sg-CDPK3 digested by *Sal* I enzymes. **b** Amplification of the 5′ and 3′ regions of eIF-5A and DHFR* cassette. (Lane M) DNA marker DL5000; (Lane 1) The amplification products of eIF-5A and CDPK3 5′ regions; (Lane 2) The amplification products of eIF-5A and CDPK3 3′ regions; 3: The amplification products of *DHFR*. **c** Identification of the homology template plasmid. (Lane M) DNA marker DL5000; (Lane 1) The amplification products of the 5′ region and *DHFR* fragment in each gene homologous template plasmid; (Lane 2) The amplification products of *DHFR* fragment and 3′ region in each gene homologous template plasmid; (Lane 3) The amplification products of the 5′ region, *DHFR* fragment and 3′ region in each gene homologous template plasmid. **d** Identification of the knockout mutant by PCR. (Lane M) DNA marker DL2000; (Lane 1) The amplification products of the 5′ region of each gene and part of the 5′ terminal of the *DHFR* fragment from gene editing or RH tachyzoites; (Lane 2) The amplification products of part of the 3′ terminal of the *DHFR* fragment and 3′ region of each gene from gene editing or RH tachyzoites; (Lane 3) The amplification products of part of the deleted genes containing the sgRNAs from gene editing or RH tachyzoites

In order to study the biological functions of TgeIF-5A, the CRISPR/Cas9 system was used as an efficient gene deletion method. In addition, given its important role in cell viability and proliferation, a reported gene CDPK3 was selected as a control to generate the knockout strains. Special sgRNAs were designed and replaced pSAG1-CAS9-TgU6-sgRNA (UPRT), which was confirmed by restriction enzyme analysis and nucleotide sequencing (Fig. 1a). To knock out eIF-5A and introduce *DHFR* resistance, peIF-5A-*DHFR* containing the *DHFR* cassettes flanked by 5′ and 3′ homologous arms was constructed. Subsequently, the plasmids were confirmed by PCR amplification (Fig. 1b, c).

To delete eIF-5A, pCRISPR-eIF-5A and peIF-5A-*DHFR* were transiently co-transfected into tachyzoites and selected with pyrimethamine. The knockout mutant was identified by PCR, and the knockout experiments were performed independently five times. Only the parasites to delete CDPK3 were identified as positive clones; no eIF-5A knockout parasite was obtained (Fig. 1d).

### Effective eIF-5A silencing by RNA interference (RNAi)

**Fig. 2:**
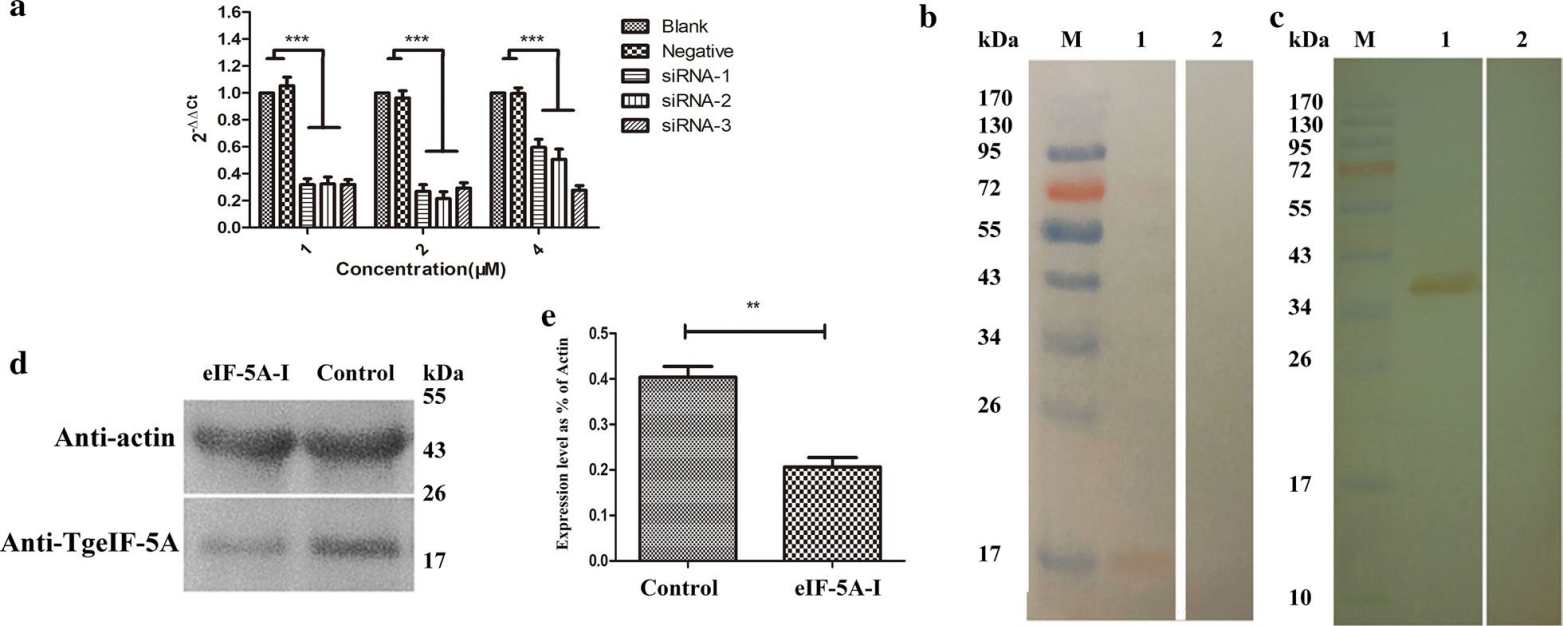
Verification of the interference effect of TgeIF-5A knockdown tachyzoites. Three specific siRNAs were transfected into tachyzoites in 1 μM, 2 μM and 4 μM, respectively. A nonspecific siRNA group was established as the control group, and a phosphate-buffered saline (PBS) group was established as a blank group. After culture for 24 h, the tachyzoites were collected. **a** Real-time PCR assessment of the knockdown of TgeIF-5A. The total RNA of the tachyzoites was extracted for reverse transcription. The relative abundance was estimated using the 2^−∆∆Ct^ method, following normalization to β-tubulin. *** *P* < 0.001. Two-way ANOVA with Bonferroni post hoc test. **b** Western blot of the total soluble protein of *T. gondii* tachyzoites. (Lane M) protein marker; (Lane 1) The total soluble protein of *T. gondii* tachyzoites probed by sera from rats immunized by rTgeIF-5A, a band at about 17 kDa was identified that was consistent with the molecular weight of native TgeIF-5A protein; (Lane 2) The total soluble protein of *T. gondii* tachyzoites probed by sera of normal rats, no specific band was identified. **c** Western blot of rTgeIF-5A. (Lane M) Protein marker; (Lane 1) Recombinant protein TgeIF-5A probed by sera from rats experimentally infected with *T. gondii* as primary antibody, a band about 36 kDa was identified that was consistent with the molecular weight of rTgeIF-5A (fused with the polyhis-tag protein of pET-32a (+) vector); (Lane 2) Recombinant protein TgeIF-5A probed by sera of normal rats as the primary antibody, no specific band was identified. **d** Western blot analysis of TgeIF-5A knockdown parasites. The total soluble proteins of TgeIF-5A knockdown and RH tachyzoites were generated. (Line eIF-5A-I) The total soluble proteins of TgeIF-5A knockdown tachyzoites were probed by antibody against β-actin and rTgeIF-5A, respectively. (Line Control) The total soluble proteins of RH tachyzoites were probed by antibody against β-actin and rTgeIF-5A, respectively. **e** Analysis of TgeIF-5A expression level in TgeIF-5A knockdown and control tachyzoites by Western blotting. The protein level of TgeIF-5A is shown as a percentage of actin in each sample. The data are indicative of three individual experiments, ** *P* < 0.01, Student’s *t* test

For the eIF-5A functional study, three specific siRNAs were designed and utilized. To check the interference efficiency and to determine the suitable siRNA for further experiments, real-time PCR and Western blotting were performed. Induction of eIF-5A knockdown resulted in a decrease in transcription (at 24 h); in particular, TgeIF-5A-siRNA-2 in 2 μM caused a 78% decrease (ANOVA: *F*_(8,73)_ = 4.26, *P* = 0.0003) (Fig. 2a).

The antibodies against TgeIF-5A were analyzed by Western blot, which showed TgeIF-5A protein with potential immunogenicity, and can be used to assess the expression of eIF-5A protein in tachyzoites treated with TgeIF-5A-siRNA-2 in 2 μM by Western blotting (Fig. 2b, c). The result revealed a parallel decrease in the expression levels of TgeIF-5A protein in tachyzoites after eIF-5A knockdown (*t* test: *t*_(4)_ = 6.254, *P* = 0.0017) (Fig. 2d, e). Consequently, 2 μM TgeIF-5A-siRNA-2 was selected to study the function of eIF-5A in further experiments.

### Overview of proteomic data

**Fig. 3:**
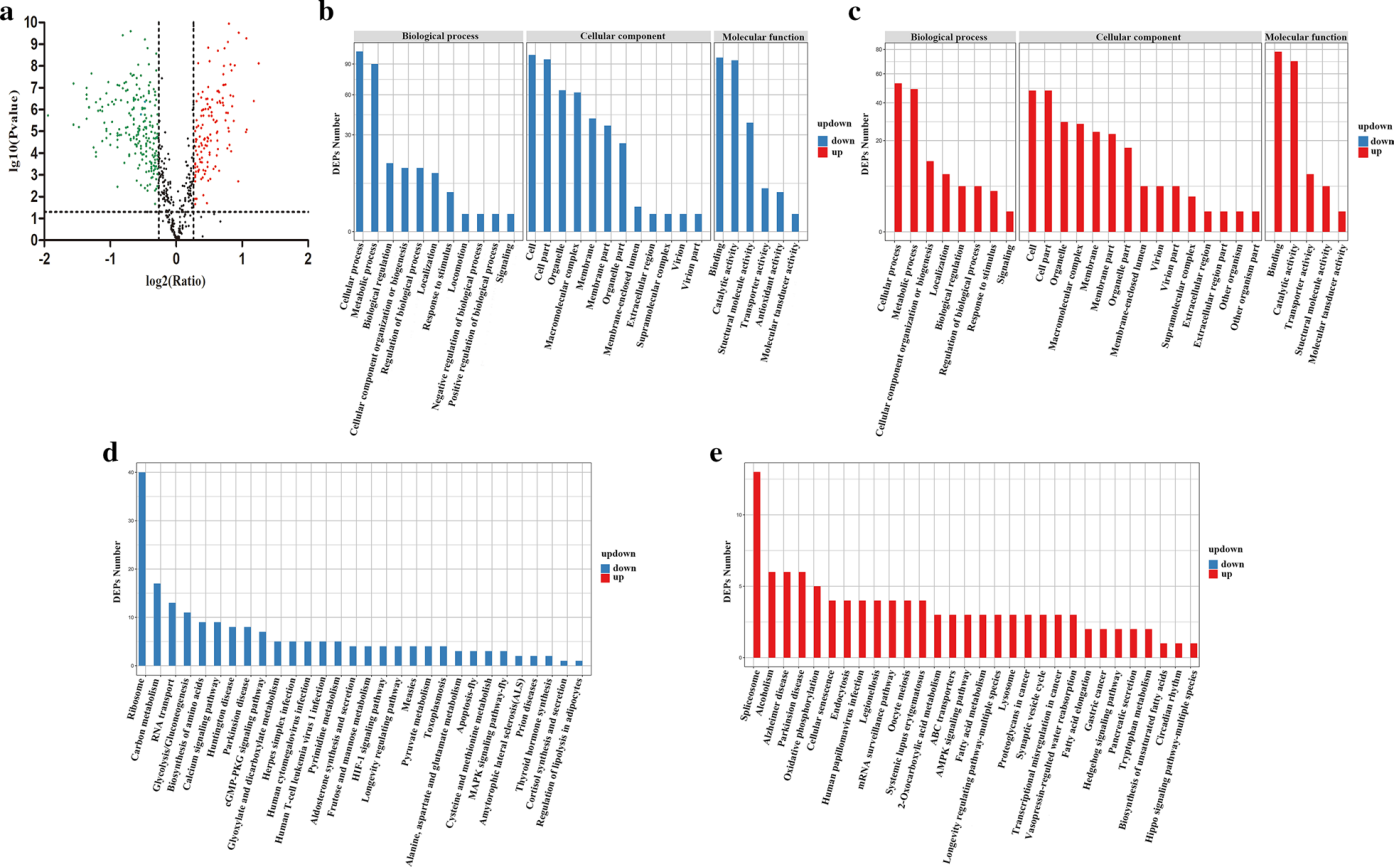
Analysis of differentially expressed proteins (DEPs). **a** Volcano plot of DEPs. This plot depicts volcano plot of log2 fold change (*x*-axis) versus -log10 *P*-value (y-axis, representing the probability that the protein is differentially expressed) of the 581 proteins identified in the iTRAQ. *P*-value < 0.05 and fold-change > 1.2 are set as the significant threshold for differential expression. The red and green dots indicate points-of-interest that display both large-magnitude fold-changes as well as high statistical significance. Dots in red indicate significantly upregulated proteins which passed the screening threshold. Dots in green indicate significantly downregulated proteins which passed the screening threshold. Black dots indicate nonsignificant DEPs. **b** GO analysis of downregulated DEPs.* X*-axis: Names of different GO terms. *Y*-axis: The number of downregulated DEPs. **c** GO analysis of upregulated DEPs. *X*-axis: Names of different GO terms. *Y*-axis: The number of upregulated DEPs. **d** KEGG analysis of downregulated DEPs. *X*-axis: Names of different KEGG pathways. *Y*-axis: The number of downregulated DEPs. **e** KEGG analysis of upregulated DEPs. *X*-axis: Names of different KEGG pathways. *Y*-axis: The number of upregulated DEPs

To assess the role of TgeIF-5A in tachyzoites, iTRAQ technology was applied to identify the proteomic differences between eIF-5A gene knockdown and RH strain parasites. A total of 581 proteins from 1391 peptides were identified with 1% false discovery rate, of which 216 proteins were identified with one unique peptide and the others were identified with at least two peptides (Additional file 5: Table S5). Volcano plots were examined to identify the proteins that were possibly responsible for the difference between eIF-5A knockdown and RH strain parasites (Fig. 3a) with a statistically significant difference (> 1.2-fold changes) (mean value of all comparison groups); *P* < 0.05(*t* test of all comparison groups). In the comparison, 359 proteins were defined as DEPs, among these 223 and 136 proteins were downregulated and upregulated, respectively.

### Analysis of the DEPs

Gene Ontology enrichment was analyzed to unify the representation of DEPs. As shown in Fig. 3b and c, the top two GO terms enriched in both downregulated and upregulated proteins for BP involved in cellular and metabolic processes were cell and cell part for CC and binding and catalytic activity for MF.

To identify the involvement of TgeIF-5A in biological pathways, pathway enrichment analysis of DEPs based on the KEGG database was performed, which showed that 140 and 82 DEPs had a KEGG Orthology (KO) ID, and could be mapped to 30 pathways in downregulated and upregulated DEPs, respectively. Among all the enriched pathways, the top five were ribosome, carbon metabolism, RNA transport, spliceosome and glycolysis/gluconeogenesis, and only the spliceosome pathway was upregulated (Fig. 3d, e). In addition, to elucidate how eIF-5A interacts with the ribosome pathway, the expression pattern of ribosomal proteins was analyzed, and showed that 40 and 5 ribosomal proteins were downregulated and upregulated, respectively (Table 1).

**Table 1:** The ribosomal proteins regulated by TgeIF-5A

Ribosomal proteins	sieIF5A-VS-RH
Ribosomal protein RPL3	Up
Ribosomal-ubiquitin protein RPL40	Up
Ribosomal protein RPS14	Up
Ribosomal protein RPS27	Up
Ribosomal protein RPL30	Up
Ribosomal protein RPS3A	Down
Ribosomal protein RPS15A	Down
Ribosomal protein RPS3	Down
Ribosomal protein RPL21	Down
Ribosomal protein RPL27A	Down
Ribosomal protein RPL12	Down
Ribosomal protein RPL27	Down
Ribosomal-ubiquitin protein RPS27A	Down
Ribosomal protein RPSA	Down
Ribosomal protein RPS4	Down
Ribosomal protein RPL7A	Down
Ribosomal protein RPL8	Down
Ribosomal protein RPS28	Down
Ribosomal protein RPL4	Down
Ribosomal protein RPL15	Down
Ribosomal protein RPL7	Down
Ribosomal protein RPS19	Down
Ribosomal protein RPL18	Down
Ribosomal protein RPL32	Down
Ribosomal protein RPL10A	Down
Ribosomal protein RPS12	Down
Ribosomal protein RPL18A	Down
Ribosomal protein RPL13	Down
Ribosomal protein RPL5	Down
Ribosomal protein RPS16	Down
Ribosomal protein RPL17	Down
Ribosomal protein RPL23A	Down
Ribosomal protein RPL36	Down
Ribosomal protein RPS25	Down
Ribosomal protein RPS8	Down
Ribosomal protein RPL13A	Down
Ribosomal protein RPL10	Down
Ribosomal protein RPS20	Down
Ribosomal protein RPL14	Down
Ribosomal protein RPP2	Down
Ribosomal protein RPL26	Down
Ribosomal protein RPL35A	Down
Ribosomal protein RPS18	Down
Ribosomal protein RPL9	Down
Ribosomal protein RPS15	Down

**Fig. 4:**
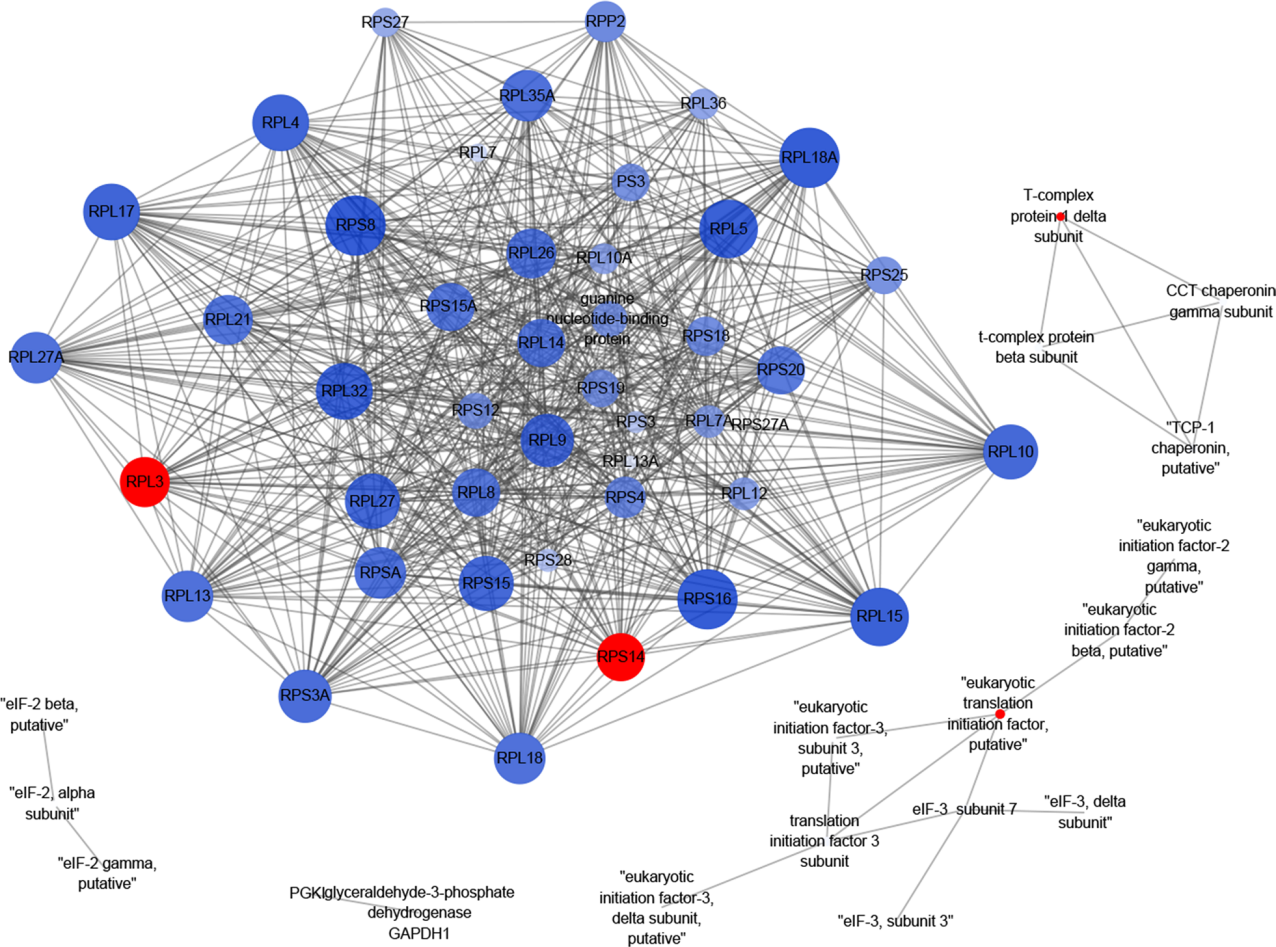
Protein–protein interactions (PPI) of DEPs identified by iTRAQ between eIF-5A knockdown tachyzoites and RH tachyzoites. The PPIs of the DEPs were identified using the STRING database (version 11.0), and the PPI networks were generated by Cytoscape software. Proteins are indicated with nodes, and the interactions between proteins are represented by edges. Blue and red nodes indicate downregulated and upregulated proteins, respectively. The size of the node indicates the interactions of DEPs: more edges (large) and fewer edges (small). The color of the node also indicates the interactions of DEPs: more edges (dark) and fewer edges (light)

Typical interactions with a combined score of 0.999 were used to generate the PPI network, and results showed that there were 62 nodes and 640 edges in the DEPs between the eIF-5A gene knockdown and RH strain parasites: five PPI clusters were included, including the main cluster of ribosomal proteins containing RPS8, RPL18A and RPS16 that had 38 edges, and a cluster of eIF-related proteins (Fig. 4).

### Analysis of variable surface protein and secreted effector expression

**Fig. 5:**
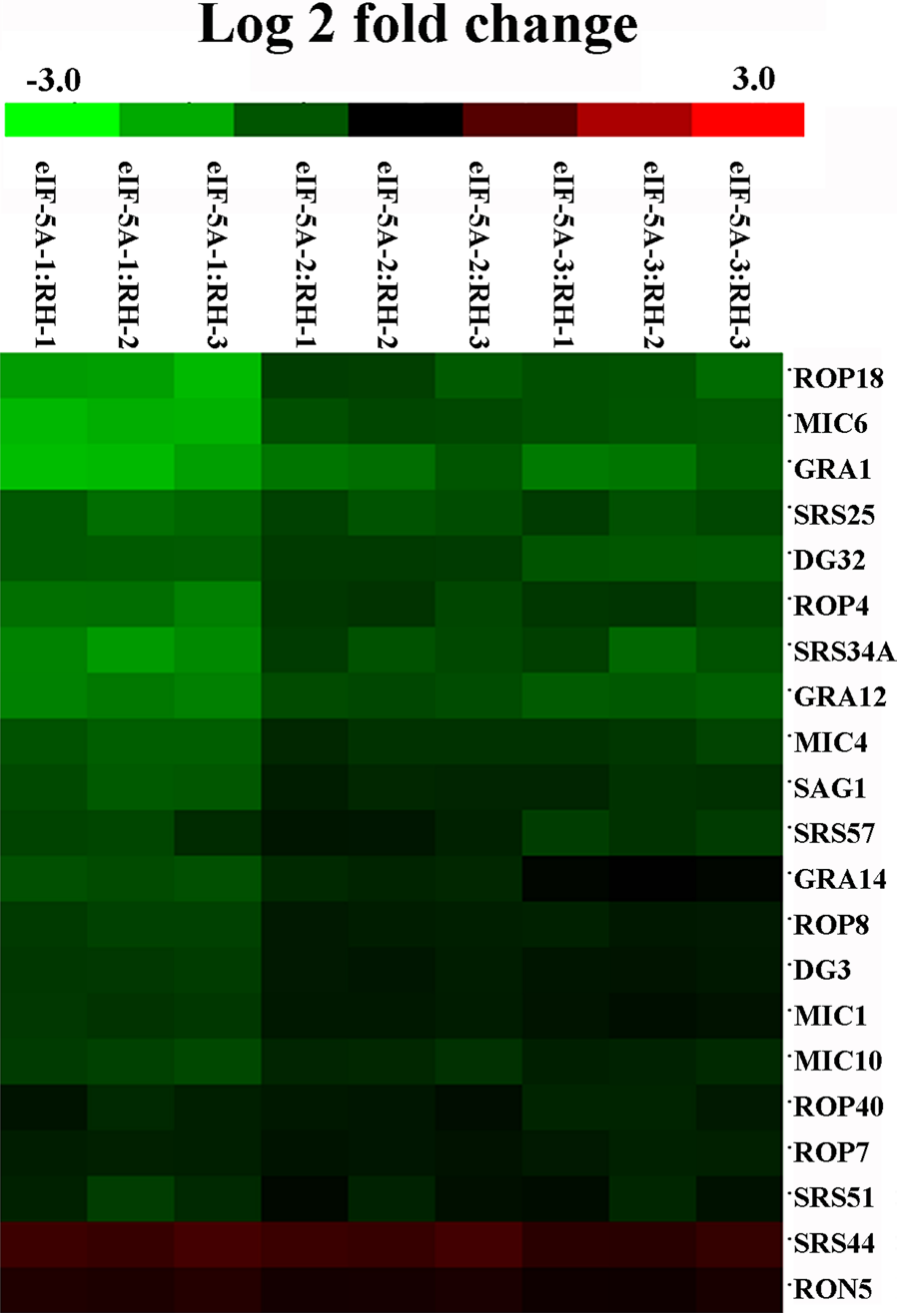
Expression profiles of differentially expressed variable surface proteins and secreted effectors. Expression values are log2-transformed, and the expression levels are annotated with a gradient color scheme. Green indicates downregulation, red indicates upregulation. Normalized protein data from different sample groups are subjected to Cluster 3.0 software and visualized using Java TreeView. Quantified proteins are grouped based on sample groups

To study the roles of TgeIF-5A in *T. gondii* tachyzoites, the expression levels of variable surface proteins and secreted effectors detected as DEPs were analyzed, which showed that 21 factors were regulated by eIF-5A, among which the content of 15 secretory organelles, micronemes, rhoptries and dense granules, and six surface antigens (SAGs) was identified. Only two proteins, SAG-related sequence (SRS44) and rhoptry neck protein 5 (RON5), were upregulated, and the other 19 factors were downregulated in eIF-5A knockdown parasites (Fig. 5 and Table 2). SAG1, five SAG-related sequences, and four MICs related to adhesion were detected, and in addition to SRS44, the other nine were downregulated. Three ROP, two rhoptry kinase family protein, one RON, and five dense granule protein alterations generally considered to be related to parasite invasion were identified, and only RON5 was upregulated.

**Table 2:** The variable surface proteins and secreted effectors regulated by TgeIF-5A

Function	Regulation	Proteins
Adhesion	Down	Microneme protein MIC1
	Down	Microneme protein MIC4
	Down	Microneme protein MIC6
	Down	Microneme protein MIC10
	Down	Surface antigen 1, SAG1
	Down	Surface antigen P22, SRS34A
	Down	Surface antigen, SRS57
	Down	SAG-related sequence SRS51
	Down	SAG-related sequence SRS25
	Up	SAG-related sequence SRS44
Invasion	Down	Rhoptry protein ROP7
	Down	Rhoptry protein ROP4
	Down	Rhoptry protein ROP8
	Down	Rhoptry kinase family protein ROP18
	Down	Rhoptry kinase family protein ROP40
	Up	Rhoptry neck protein RON5
	Down	Dense granule protein GRA14
	Down	Dense granule protein 3, GRA3
	Down	Dense granule protein GRA12
	Down	Dense-granule antigen DG32 (GRA32)
	Down	Dense granule protein 1, GRA1

### eIF-5A participated in the invasion process of tachyzoites

**Fig. 6:**
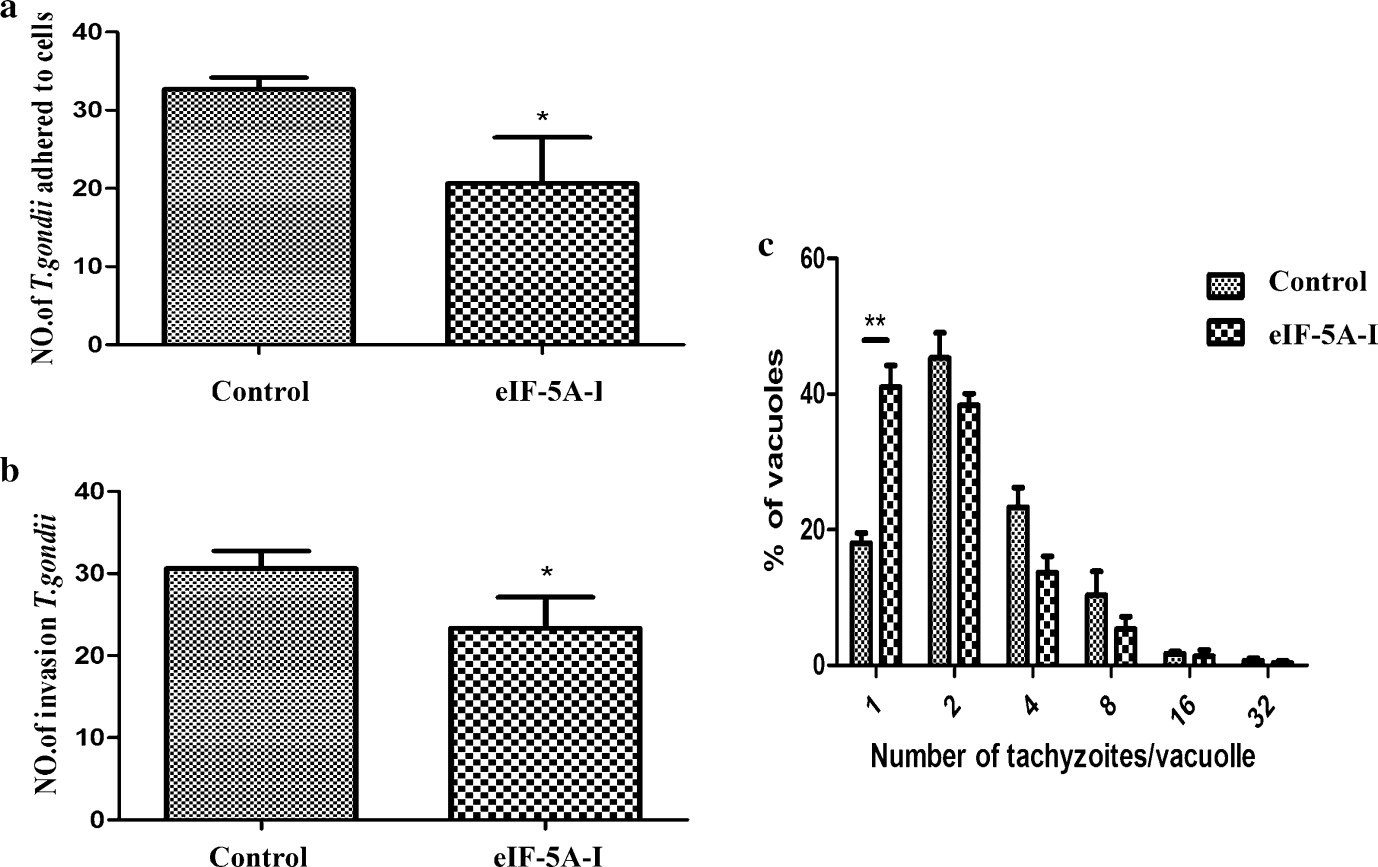
Analysis of the invasion and replication ability of eIF-5A knockdown tachyzoites. **a** Attachment ability of eIF-5A knockdown parasites. RH and eIF-5A knockdown parasites were inoculated in the fixed monolayer cells for 1 h, and immunofluorescence assay (IFA) was performed, after which the number of parasites adhered to host cells was counted. The data are indicative of three individual experiments, * *P* < 0.05, Student’s *t* test. **b** Invasion ability of eIF-5A knockdown parasites. RH and eIF-5A knockdown parasites were inoculated in HFF cells for 1 h, and IFA was performed, after which the number of parasites invading the cells was counted. The data are indicative of three individual experiments, * *P* < 0.05, Student’s *t* test. **c** Replication ability of eIF-5A knockdown tachyzoites. RH and eIF-5A knockdown parasites were inoculated in HFF cells for 24 h, and the parasite number of 100 parasitophorous vacuoles was counted and analyzed using Student’s *t* test with GraphPad Prism 5.0 (GraphPad Software, USA). The data are indicative of three individual experiments (** *P* < 0.01)

To verify the role of TgeIF-5A in parasite invasion, the ability of eIF-5A knockdown strains to adhere and invade host cells was checked by immunofluorescence assay. The results revealed that TgeIF-5A knockdown parasites displayed significantly reduced adhering efficiency (*t* test: *t*_(4)_ = 3.432, *P* = 0.0265) (Fig. 6a), and as shown in Fig. 6b, the parasites invaded into host cells were also significantly reduced (*t* test: *t*_(4)_ = 3.101, *P* = 0.0362).

### eIF-5A involved in the replication of tachyzoites

To demonstrate whether TgeIF-5A played any role during tachyzoite replication, the ability of TgeIF-5A in replication of tachyzoites in vitro was estimated by counting the number of parasites contained in 100 PVs 24 h after invasion. In eIF-5A knockdown parasites, the percentage of PVs containing one tachyzoite increased to 43%, compared to 18% in the wild RH strain (*t* test: *t*_(4)_ = 6.462, *P* = 0.0030), and the number of all other PVs containing more than one tachyzoite decreased, suggesting that TgeIF-5A was involved in tachyzoite replication (Fig. 6c).

### Growth of eIF-5A gene knockdown parasites in vitro and in vivo

**Fig. 7:**
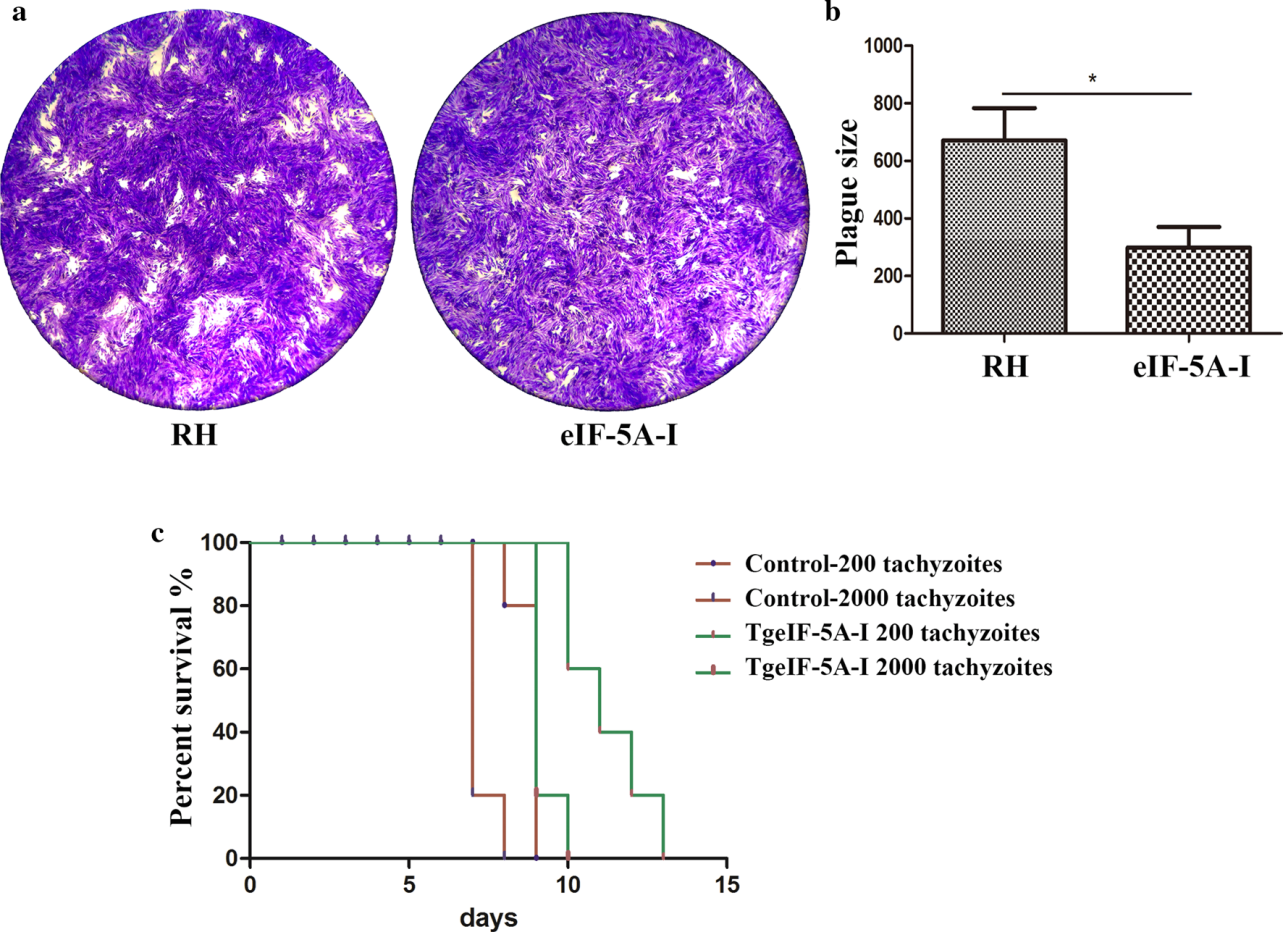
Growth of eIF-5A knockdown and RH parasites in vitro and in vivo. **a** Plaque assay in HFF cells. RH and eIF-5A knockdown parasites were inoculated in HFF cells, and plaques are visible as clear zones against the background of crystal violet-stained HFF monolayers. **b** Size of the plaques. The size of plaques was evaluated by Image Pro Plus 6.0 (Media Cybernetics, USA), and the data were analyzed by Student’s *t *test using GraphPad Prism 5.0 (GraphPad Software, USA). The data were indicative of three individual experiments (* *P* < 0.05). **c** Virulence analysis of eIF-5A knockdown parasites in BALB/c mice. Two doses of RH and eIF-5A knockdown parasites were injected into mice (200 or 2000 parasites/mouse and 5 mice/dose, respectively). The survival time was monitored each day, and the significance of each dose was compared by the log-rank test and Gehan Breslow Wilcoxon test (*** *P* < 0.001)

To assess whether eIF-5A played any role in growth, parasites were allowed to form plaques on HFF monolayers; after culture for 7 days, the formation of plaques was assessed, which showed that the plaques produced by TgeIF-5A gene knockdown tachyzoites were fewer and smaller than those in the control groups (*t* test: *t*_(4)_ = 2.784, *P* = 0.0496) (Fig. 7a, b).

Additionally, an animal experiment was performed. Parasites after transfection with siRNA were intraperitoneally injected into BALB/c female mice, which showed that the survival time of mice infected with eIF-5A gene knockdown parasites was significantly prolonged compared to the groups infected with the control parasites in both infectious doses (log-rank (Mantel-Cox) test: *χ*^2^ = 38.99, *df* = 3*, P* < 0.0001) (Fig. 7c).

## Discussion

Originally known as an initiation factor, eIF-5A is unusual in contributing to both translation and elongation [28], and its functions in cell biology, development and oncology are active topics of research [29, 30]. Among these functions are translational controls related to selective translation of specific mRNAs, which further promotes cell proliferation and is involved in the pathogenicity of several cancer types [28, 31]. Over-expression of eIF-5A has often shown a strong correlation with cancer and has been considered as a candidate oncogene [32, 33]. Meanwhile, studies on parasites showed that eIF-5A participated in the developmental stages (trophozoites) of *Plasmodium vivax*, suggesting its vital role in proliferation [34]. In *Trypanosoma brucei*, eIF-5A was involved in the translation of proteins for abnormal cell morphology and detached flagella [35]. Based on previous studies considering eIF-5A as a selective target of therapeutic interest, in this study we verified that eIF-5A plays an important role in the growth and replication of *T. gondii* tachyzoites. The most efficient gene editing tool, CRISPR/Cas9, was used to generate an eIF-5A knockout mutant. After many repetitions, the surviving parasites continued to express eIF-5A, while the control gene was easily generated, suggesting that eIF-5A is essential for *T. gondii* tachyzoites.

To obtain additional insight into the function of eIF-5A in *T. gondii*, a TgeIF-5A knockdown tachyzoite was generated by RNAi, which showed effective inhibition in both transcription and protein levels. The iTRAQ and bioinformatics were used to identify significant proteins regulated by TgeIF-5A. A total of 581 proteins were identified, which is a lower number than reported in previous studies [36, 37]. This might be related to the lower number of tachyzoites used and the treatment of tachyzoites with electroporation in this study. In the pathway analysis of DEPs, ribosomes, RNA transport and spliceosomes were three significantly regulated pathways. RNA transport is a key determinant in the spatiotemporal articulation of gene expression, and pre-mRNA conversion to mRNA by splicing is an essential step in gene expression, in which intron deletion and exon ligation together occur by spliceosomes [38, 39]. Ribosomes catalyze the translation of genetic information of mRNA to synthesize proteins [40]. Consistent with previous studies, we identified 45 ribosomal proteins, among which TgeIF-5A was indeed an active protein participating in the gene expression of *T. gondii*, especially in the translation phase [28].

*T. gondii* is an obligate intracellular parasite in which a strong host cell attachment is established through MICs and SAGs. Previous studies demonstrated that multi-adhesive complexes such as TgMIC1/4/6 play a critical role in host cell attachment, in which MIC4 performs host cell binding activity, MIC6 interacts with aldolase and the parasite cytoskeleton, and the architecture of MIC1 provides the basis for subsequent trafficking of the complex to the micronemes [41, 42]. It was further suggested that SAG1 and SAG3 mediate parasite adherence to glycoproteins and proteoglycans on the host cell membrane, respectively [43, 44]. Upon contact with an appropriate cell, tachyzoites can invade within seconds, without apparent disruption to the invaded cell [45]. In this study, we evaluated the potential role of eIF-5A through analysis of DEPs, in which four MICs were downregulated, including the identified multi-adhesive complexes TgMIC1/4/6 [46]. Five SAGs were downregulated as well. In addition, we performed in vitro studies to evaluate the ability of tachyzoites to attach to host cells, and the results showed that after inhibition of TgeIF-5A, the number of tachyzoites adhering to cells was significantly reduced. Taken together, these results demonstrate that eIF-5A promotes efficient parasite attachment by regulating the expression of adhesions.

Once the parasite had attached securely to the host cell, the secretion of rhoptries followed rapidly, leading to a decrease in host cell viscosity, initiating invagination and enhancing invasion [47]. ROPs have been suggested to be involved in building the moving junction and the formation of PV [48]. In our current study, six rhoptries were included in the DEPs, of which ROP4 was located in both rhoptries and PV membranes (PVM), and might be regulated by the protein phosphorylation machinery of the host cell [49]. The other three ROPs detected in this study, i.e. ROP7, ROP8 and ROP 18, together with ROP4 were members of the ROP2 family [50, 51]. After secretion, ROP 18 traffics to the PVM, and associates with this membrane for the whole intracellular cycle [51]. However, during and after invasion into PV, GRAs were subjected to exocytosis, which ultimately participated in the modification of the PV [52]. In this study, five GRAs were detected as DEPs; among these, GRA3 was associated with PVM and caused the PVM to protrude into the cytoplasm [53], and GRA12 was involved in the intravacuolar membranous nanotubular network [54]. Among the 11 proteins associated with the invasion process, only RON5 was upregulated, and formed the tight junction with AMA1. The ability for attachment and regulation and rhoptry secretion in AMA1 knockout parasites was impaired [55]. Nevertheless, our study highlighted that inhibition of eIF-5A significantly reduced the number of invasive tachyzoites, making the relationship between RON5 and other ROPs a hot topic of research, and this interaction is worthy of further study.

The rate of *T. gondii* replication is the major parameter defining parasite virulence [56]. The expression of variable surface proteins and secreted effectors packed into specific organelles such as rhoptries, micronemes and dense granules reflects the replication of parasites. Among rhoptry proteins, ROP2 is a large protein family including ROP2, ROP4, ROP5, ROP7, ROP8 and ROP18 [57]. Most MICs are adhesions proteins, such as MIC1, MIC4 and MIC6 [58]. Dense granular proteins are exposed to exocytosis during or after host cell invasion [59]. For instance, ROP18 is a crucial factor for controlling the replication of *T. gondii* [60]. MIC1 and GRA3 are associated with the virulence of *T. gondii *in vivo [58, 61]. In this study, proteomic results showed that 19 variable surface proteins and secreted effectors were downregulated in eIF-5A gene knockdown tachyzoites. Analysis of the DEPs revealed that the ribosomal pathway was the chief pathway regulated by eIF-5A, which is a complex, dynamic molecular machine responsible for protein synthesis. Furthermore, we performed replication and growth assay, which showed that the intracellular replication of tachyzoites and the growth in both in vivo and in vitro assays were significantly reduced after eIF-5A gene knockdown. These results suggest that eIF-5A is involved in *T. gondii* replication by regulating the expression of variable surface proteins and secreted effectors through the ribosome pathway.

## Conclusions

All the findings reported here indicate that the eIF-5A proto-oncogene influences *T. gondii* tachyzoite growth and invasion by regulating the expression of MICs, ROPs and SAGs. In addition, TgeIF-5A participates in parasite replication regulation mainly through the ribosome pathway. Importantly, these findings provide insight into the biological function of eIF-5A during invasion and growth of *T. gondii* tachyzoites, which will lead to the development of an effective vaccine or drug target against toxoplasmosis.

## Supplementary Information

**Additional file 1: Table S1.** Primer sequences for PCR amplification. The primer sequences used for PCR amplification of eIF-5A open reading frame.

**Additional file 2: Table S2.** Primer sequences for gene knockout. The primer sequences used for eIF-5A and the control gene CDPK3 knockout.

**Additional file 3: Table S3.** siRNA sequences for gene knockdown. The primer sequences used in eIF-5A siRNA assay.

**Additional file 4: Table S4.** Primer sequences for real-time PCR. The primer sequences used to verify the effect of eIF-5A knockdown.

**Additional file 5: Table S5.** Protein Identification Overview

**Additional file 6: Method S1.** The real-time PCR reactions and conditions.

**Additional file 7: Method S2.** The Western blotting assay.

## Data Availability

The mass spectrometry proteomics data have been deposited to the ProteomeXchange Consortium via the PRIDE partner repository with the dataset identifier PXD016810.

## Abbreviations

*T. gondii*: *Toxoplasma gondii*
ROP: Rhoptry protein
MIC: Microneme protein
GRA: Dense granule antigens
eEF-2: Eukaryotic elongation factor-2
PV: Parasitophorous vacuole
eIF-5A: Eukaryotic translation initiation factor 5A
DHS: Deoxyhypusine synthase
HFFs: Human foreskin fibroblasts
SD: Sprague Dawley
SPF: Specific-pathogen-free
ORF: Open reading frame
iTRAQ: Isobaric tag for relative and absolute quantitation
TEAB: Triethylammonium bicarbonate
FDR: False discovery rate
DEPs: Differentially expressed proteins
GO: Gene Ontology
CC: Cellular component
BP: Biological process
PPI: Protein–protein interaction
ANOVA: One-way analysis of variance
KEGG: Kyoto Encyclopedia of Genes and Genomes
