# Supplementary material for: Proteomics analysis reveals that the proto-oncogene eIF-5A indirectly influences the growth, invasion and replication of Toxoplasma gondii tachyzoite
Source: Parasit Vectors. 2021 May 26;14:283. doi: 10.1186/s13071-021-04791-6 (PMC8157420; doi:10.1186/s13071-021-04791-6)
Supplement: Supplementary file 1 — Additional file 1: Table S1. Primer sequences for PCR amplification. The primer sequences used for PCR amplification of eIF-5A open reading frame. [file 13071_2021_4791_MOESM1_ESM.docx]

**Table S1. Primer sequences for PCR amplification**

| Primers | Sequence |
| --- | --- |
| eIF-5A-F | CCGGAATTC ATGAGTGACGCCGAGGATGT |
| eIF-5A-R | CCCAAGCTT AGTTTGTAGCATCCTTACGCCT |
