# Supplementary material for: Proteomics analysis reveals that the proto-oncogene eIF-5A indirectly influences the growth, invasion and replication of Toxoplasma gondii tachyzoite
Source: Parasit Vectors. 2021 May 26;14:283. doi: 10.1186/s13071-021-04791-6 (PMC8157420; doi:10.1186/s13071-021-04791-6)
Supplement: Supplementary file 2 — Additional file 2: Table S2. Primer sequences for gene knockout. The primer sequences used for eIF-5A and the control gene CDPK3 knockout. [file 13071_2021_4791_MOESM2_ESM.docx]

**Table S2. Primer sequences for gene knockout**

| Primers | Sequence |
| --- | --- |
| CRISPR-eIF-5A-F | GGATGTCACCTTCGAAACCGGTTTTAGAGCTAGAAATAGC |
| CRISPR-CDPK3-F | GAGCAAGCTGACGACTCTGGGTTTTAGAGCTAGAAATAGC |
| CRISPR-R | AACTTGACATCCCCATTTAC |
| 5’-eIF-5A-F | GAGCTCGGTACCCGGGGATCCGGCTGGGTATGTGTATCGTTCTTCG |
| 5’-eIF-5A-R | TTTACAGCCTGGCGAAGCTTTTTGACGAAAAACCACCAACGACCT |
| 3’-eIF-5A-F | GCACTTGCAGGATGAATTCCTAGAGAAGGAAACACCCAGGGAATG |
| 3’-eIF-5A-R | GACCATGATTACGCCAAGCTTAGTTCTCTTAGCACCACTGTGACCG |
| 5’-CDPK3-F | GGTACCCGGGGATCCTCTAGAACGAAATCAGCGAAGACTCCCAT |
| 5’-CDPK3-R | TTTACAGCCTGGCGAAGCTTTCAAATCCAGGCTTCAGATCGTC |
| 3’-CDPK3-F | GCACTTGCAGGATGAATTCCCCTCTCGGCGATTCTGATTCTC |
| 3’-CDPK3-R | GACCATGATTACGCCAAGCTTAAGATACACGAGTCCAGATTCAGCG |
| DHFR-F | AAGCTTCGCCAGGCTGTAAATCC |
| DHFR-R | GGAATTCATCCTGCAAGTGCATAGAAG |
| Y-eIF1-F | AGAGAACTCAGGGGTGCCAG |
| Y-eIF2-R | CCTGCGCCTAACAGTCCTAA |
| Y-eIF3-F | GGAGCGATCAAGAAGAACGGTT |
| Y-eIF3-R | CAGCTCCTTTGAGGCGATGATT |
| Y-CDPK3-1-F | TGTGTCGTGAACTCCGTGCT |
| Y-CDPK3-2-R | TGCTTGAGAGAGCCTGTTTCC |
| Y-CDPK3-3-F | CCCATGACTCCAGGCATGTA |
| Y-CDPK3-3-R | CTGTTGGAAGGCAGCAAGAAG |
| Y-PCR1-R | TACCAGTCATGGACGAGATCG |
| Y-PCR2-F | ACACGCATGTCTACACGAACC |
