# Supplementary material for: Proteomics analysis reveals that the proto-oncogene eIF-5A indirectly influences the growth, invasion and replication of Toxoplasma gondii tachyzoite
Source: Parasit Vectors. 2021 May 26;14:283. doi: 10.1186/s13071-021-04791-6 (PMC8157420; doi:10.1186/s13071-021-04791-6)
Supplement: Supplementary file 3 — Additional file 3: Table S3. siRNA sequences for gene knockdown. The primer sequences used in eIF-5A siRNA assay. [file 13071_2021_4791_MOESM3_ESM.docx]

**Table S3. siRNA sequences for gene knockdown**

| Primers | Sequence |
| --- | --- |
| eIF-5A-siRNA1 | ACGUCCACUGUUGUAUACAUCAGUA  UACUGAUGUAUACAACAGUGGACGU |
| eIF-5A-siRNA2 | CCACUACACCAUCUUCCCUACUUUA  UAAAGUAGGGAAGAUGGUGUAGUGG |
|  |  |
| eIF-5A-siRNA3 | CAAUCAGGUUCAUACGUCCACUGUU  AACAGUGGACGUAUGAACCUGAUUG |
