# Supplementary material for: Proteomics analysis reveals that the proto-oncogene eIF-5A indirectly influences the growth, invasion and replication of Toxoplasma gondii tachyzoite
Source: Parasit Vectors. 2021 May 26;14:283. doi: 10.1186/s13071-021-04791-6 (PMC8157420; doi:10.1186/s13071-021-04791-6)
Supplement: Supplementary file 4 — Additional file 4: Table S4. Primer sequences for real-time PCR. The primer sequences used to verify the effect of eIF-5A knockdown. [file 13071_2021_4791_MOESM4_ESM.docx]

**Table S4. Primer sequences for real-time PCR**

| Primers | Sequence |
| --- | --- |
| RT-eIF-5A-1-F | GCGTCCCACACATATCCCAT |
| RT-eIF-5A-1-R | TGCTTTCCGGTTTTGGAGGT |
| RT-eIF-5A-2-F | TTGCCCGACTTCTCACAACA |
| RT-eIF-5A-2-R | AGTCCTTGGGCAGCATCAAA |
| RT-eIF-5A-3-F | GAAAGCATGGTCACGCCAAG |
| RT-eIF-5A-3-R | TGTGAGAAGTCGGGCAAACA |
| RT-eIF-5A-4-F | TTTGATGCTGCCCAAGGACT |
| RT-eIF-5A-4-R | GTCACAAGAACCGACTTGCC |
| RT-eIF-5A-5-F | TGCCCGACTTCTCACAACAT |
| RT-eIF-5A-5-R | GCAGAAGCCATCATCGGACA |
| RT-β-tubulin-F | AAATCGTCCACGTTCAGGGT |
| RT-β-tubulin-R | AGCTGCAAGTCACTGTCTCC |
