# Supplementary material for: Proteomics analysis reveals that the proto-oncogene eIF-5A indirectly influences the growth, invasion and replication of Toxoplasma gondii tachyzoite
Source: Parasit Vectors. 2021 May 26;14:283. doi: 10.1186/s13071-021-04791-6 (PMC8157420; doi:10.1186/s13071-021-04791-6)
Supplement: Supplementary file 5 — Additional file 5: Table S5. Protein Identification Overview [file 13071_2021_4791_MOESM5_ESM.docx]

**Table S5. Protein Identification Overview**

| Sample name | Total spectra | Spectra | Unique Spetra | Peptide | Unique Peptide | Protein |
| --- | --- | --- | --- | --- | --- | --- |
| *Toxoplasma gondii* | 355152 | 3580 | 3534 | 1391 | 1377 | 581 |
