# Supplementary material for: Proteomics analysis reveals that the proto-oncogene eIF-5A indirectly influences the growth, invasion and replication of Toxoplasma gondii tachyzoite
Source: Parasit Vectors. 2021 May 26;14:283. doi: 10.1186/s13071-021-04791-6 (PMC8157420; doi:10.1186/s13071-021-04791-6)
Supplement: Supplementary file 6 — Additional file 6: Method S1. The real-time PCR reactions and conditions. [file 13071_2021_4791_MOESM6_ESM.docx]

**Additional file 1: Method S1. The real-time PCR reactions and conditions**

The real-time PCR reactions were carried out in 96-well optical reaction plates (Bio-Rad, USA) with 4 nM of each specific primer (Table S4), 2 μl cDNA, and SYBR® Green Master Mix (Vazyme, China) using an ABI 7500 Real-Time PCR system (Applied Biosystems, USA). The PCR conditions were as follows: 95°C for 30 sec, followed by 95°C for 10 sec, 60°C for 30 sec for 40 cycles, then 95°C for 15 sec, 60°C for 60 sec, and 95°C for 15 sec. The relative abundance of the transcript in each group was estimated using the 2^-∆∆Ct^ method, following normalization to β-tubulin.
