# Supplementary material for: Proteomics analysis reveals that the proto-oncogene eIF-5A indirectly influences the growth, invasion and replication of Toxoplasma gondii tachyzoite
Source: Parasit Vectors. 2021 May 26;14:283. doi: 10.1186/s13071-021-04791-6 (PMC8157420; doi:10.1186/s13071-021-04791-6)
Supplement: Supplementary file 7 — Additional file 7: Method S2. The Western blotting assay. [file 13071_2021_4791_MOESM7_ESM.docx]

**Method S2. The Western blotting assay**

The total soluble protein of *T. gondii* tachyzoites was obtained as described previously [1], and isolated by 10% SDS-PAGE then the proteins were moved to polyvinylidene fluoride (Millipore, USA). The polyclonal antibodies against TgeIF-5A were used for immunoblotting analysis. Finally, a Pierce™ ECL Western Blotting Substrate Kit was utilized to identify the bands.

**Reference:**

1. **Liu X, Ma Q, Sun X, Lu M, Ehsan M, Hasan MW, Xu L, Yan RF, Song XK, Li XR.** 2017. Effects of Recombinant Toxoplasma gondii Citrate Synthase I on the Cellular Functions of Murine Macrophages In vitro. Frontiers in microbiology **8:**1376.
